# Evaluation of Loco-Regional Skin Toxicity Induced by an In Situ Forming Depot after a Single Subcutaneous Injection at Different Volumes and Flow Rates in Göttingen Minipigs

**DOI:** 10.3390/ijms22179250

**Published:** 2021-08-26

**Authors:** Charlotte Peloso, Anne-Pascale Trichet, Jacques Descotes, Joël Richard, Christophe Roberge, Adolfo Lopez-Noriega

**Affiliations:** 1 MedinCell S.A., 3 rue des Frères Lumière, 34830 Jacou, France; charlotte.peloso@medincell.com (C.P.); annepascale.trichet@medincell.com (A.-P.T.); joel.richard@medincell.com (J.R.); christophe.roberge@medincell.com (C.R.); 2 ImmunoSafe Consultance, 120 Chemin des Bouleaux, 38480 Saint-Jean-d’Avelanne, France; jacques.descotes@medincell.com

**Keywords:** loco-regional skin tolerability, in situ forming depots, subcutaneous injection, flow rate, minipig, sustained release drug delivery

## Abstract

The present study aims to investigate the loco-regional tolerability and injection parameters (i.e., flow rate and administration volume) of an in situ forming depot (ISFD) in Göttingen minipigs, to secure both the therapeutic procedure and compliance in chronic medical prescriptions. The ISFD BEPO^®^ technology (MedinCell S.A.) is investigated over 10 days, after a single subcutaneous injection of test item based on a DMSO solution of diblock and triblock polyethylene glycol-polylactic acid copolymers. Injection sites are systematically observed for macroscopic loco-regional skin reactions as well as ultrasound scanning, enabling longitudinal in vivo imaging of the depot. Observations are complemented by histopathological examinations at 72 h and 240 h post-injection. Overall, no treatment-emergent adverse effects are macroscopically or microscopically observed at the subcutaneous injection sites, for the tested injection flow rates of 1 and 8 mL/min and volumes of 0.2 and 1 mL. The histopathology examination confirms an expected foreign body reaction, with an intensity depending on the injected volume. The depot morphology is similar irrespective of the administration flow rates. These results indicate that the ISFD BEPO^®^ technology can be considered safe when administered subcutaneously in Göttingen minipigs, a human-relevant animal model for subcutaneous administrations, in the tested ranges.

## 1. Introduction

Over the past few decades, the field of smart and controlled delivery systems has been continuously expanding, investigating approaches from the nano to the macrometric scale [1] and from nondegradable implants to bioresorbable systems [2,3]. Most of these drug delivery strategies are designed to improve the bioavailability and pharmacokinetics of target therapeutic molecules, with a view to reducing dosing frequency compared to immediate release dosage forms and, consequently, improving treatment compliance [4]. Among the available formulation approaches, in situ forming depots (ISFD) are particularly attractive as they are designed to bio-resorb and are often easier to administer compared to preformed delivery systems [5]. The common feature of ISFD is the formation of a solid depot encapsulating a drug upon administration [5,6]. They are classified according to the solidification mechanism, i.e., in situ cross-linking [7,8], in situ solidifying organogels [9] or in situ phase separation [10,11].

Despite the growing interest of the scientific community, little is known regarding the loco-regional toxicity potential of ISFD [5,12], even though this is a key development issue of every sustained-release injectable drug. Poorly tolerated injectable formulations (e.g., local pain, discomfort, redness) may lead to a significantly lowered observance of the treatment and its discontinuation, regardless of efficacy [13,14]. In addition, repeated administrations of the product could be compromised if sensitization induces a loco-regional reaction at the injection site (e.g., itching, skin eruption). Interestingly, in addition to the biocompatibility of the formulation components, factors linked to the injection procedure are being increasingly evaluated for their influence on loco-regional tolerability, in particular, with classic aqueous-based injectable products, such as insulin or heparin. Critical factors include the type of device, the selected injection site and the injection flow rate [15,16,17]. Similar investigations are necessary with ISFD systems to demonstrate their safe use for the patient and understand if the injection criteria, particularly the injection flow rate, influences the formation of the depot and subsequent loco-regional tolerability [18,19].

MedinCell proprietary ISFD technology [20], trademarked as BEPO^®^, is composed of (1) a mixture of one diblock and one triblock copolymer of poly(ethylene glycol) (PEG) and amorphous poly(D,L-lactic acid) (PDLLA), (2) a biocompatible organic solvent and (3) an active pharmaceutical ingredient (API). The API may be either in solution or in suspension in the polymeric vehicle, which is designed to be water insoluble. Upon administration, the solvent will diffuse out of the system and be replaced by bodily fluids, causing the copolymers to precipitate and form a solid depot, which physically entraps the API. The therapeutic agent will be released by a combination of diffusion through the polymeric matrix and progressive degradation of the copolymers. BEPO^®^ technology allows an unprecedented flexibility in the control of the release, which may span from days to months by tailoring its composition [21,22], such as e.g., the size of the copolymers, the solvent type or the ratio among the formulation components. This ISFD technology is currently undergoing advanced clinical trials for both systemic drug delivery using the subcutaneous (SC) route, and local delivery within the intra-articular space.

The present article depicts the results from an experimental study designed to assess the loco-regional skin tolerability of the ISFD BEPO^®^ technology. Different volumes of a model BEPO^®^ vehicle (copolymer mixture dissolved in solvent, without API) were administered subcutaneously to Göttingen minipigs at different flow rates. The minipig was selected as it has been shown that its SC space is similar to that of humans in terms of tissue structure and biomechanics [23,24]. The test item selected for this study was composed of hydrophobic copolymers with a relatively high molecular weight (10–12 kDa). The resulting product with a high copolymer concentration in dimethyl sulfoxide (DMSO) was expected to reach a very high dynamic viscosity (>500 mPa.s). This polymeric composition was designed to give relatively slow degradation kinetics, allowing a monitoring and comparison of the different reactions with minimal variations in the depot polymeric composition during the study period. Typical injection speed for a marketed product being around 3–6 mL/min (administration of 0.5 mL to 1 mL in 10 s) [25], a wide range of manual injection speeds were selected, with a slow and a fast injection rate targeting 1 mL/min and 10 mL/min. In addition to the flow rate, two volumes of test item (i.e., 0.2 and 1 mL) were injected to assess a potential effect of increased depot volume on injection site tolerability [26,27] and depot morphology in the SC [28]. Loco-regional tolerability was assessed over 10 days by macroscopic observation and subsequent histopathology evaluation of the injection sites. It is assumed that intolerance due to the administration procedure would be noticeable within less than two weeks. Ultrasound imaging was used to complete the evaluation by providing information on the morphology of the depot and the surrounding tissues.

## 2. Results

### 2.1. Characterization of the Test Item

Test item characterization was performed immediately following batch preparation. Table 1 presents the viscosity values of the test item and the injection force needed at both flow rates (*n* = 3) as well as the Endotoxin level in the prepared batch.

In the tested shear rate range, the test item displayed a Newtonian behavior with a constant viscosity at each point measured (see Figure S1). Hence, the calculated viscosity value is the mean viscosity measured at each shear rate test time-point. The test item was confirmed to have a high viscosity, i.e., above 600 mPa.s. Regarding injectability, the difference between the injection force values at the two tested flow rates was statistically significant (*p* < 0.0001, *n* = 6). The injection force at 1 mL/min was of 2.5 N compared to 19 N at 10 mL/min. Both injection force values were below 20 N, which is considered the limit for a comfortable manual administration of an injectable drug product [29]. Thus, the experimental conditions were considered appropriate for in vivo injection. In addition, endotoxin levels were measured below 2.339 EU/mL based on the highest injected volume (i.e., 1 mL). This result is significantly below the determined limit and comply with the USP-calculated limit, confirming the low pyrogenic potential of the prepared product.

### 2.2. In Vivo Administration of the Test Item

Each animal received the full scheduled dose at each injection site. Injection durations were recorded and flow rates calculated according to the volume administered (0.2 or 1 mL). Table 2 summarizes the mean flow rates and the corresponding deviation from the target value for each treatment group.

Slow injections (1 mL/min) were performed as planned. No deviation from the targeted injection duration was observed in any of the groups. In contrast, substantial deviations were observed for the highest flow rate (10 mL/min). As a result, the maximum injection flow rate experimentally reached in this study was 8 mL/min.

### 2.3. Macroscopic Observation of Loco-Regional Skin Tolerance

**Figure 1:**
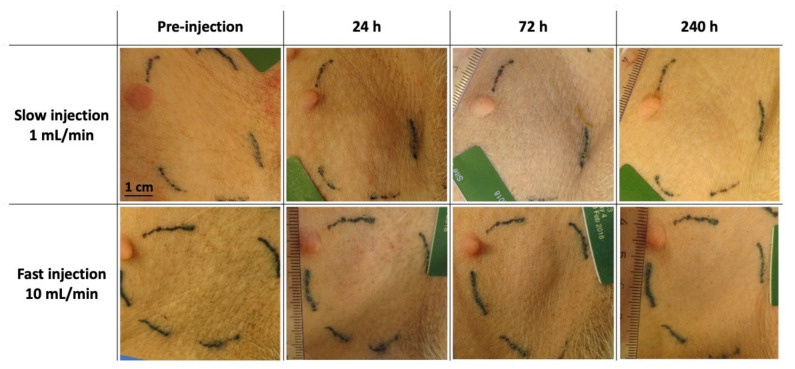
Macroscopic loco-regional tolerance assessment. Representative images of injection sites from 1 mL treated group along the study.

No treatment-linked loco-regional adverse effects were observed in the animals, regardless of the test item volume and the injection flow rate. Figure 1 is a compilation of representative pictures from both injection sites for a 1 mL-treated animal during the 10-day follow-up of the study. Representative pictures of the 0.2 mL treated group are presented in Figure S2.

From 24 h post-injection, both groups displayed a localized induration which persisted over the 10-day observation period.

### 2.4. Ultrasound Imaging

**Figure 2:**
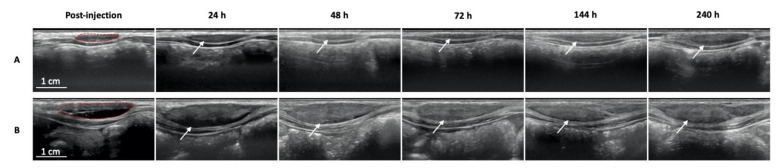
Longitudinal ultrasound imaging of (**A**) 0.2 mL and (**B**) 1 mL test item injection site over the course of the study. Images were acquired between 13 and 15 MHz along the sagittal plane, parallel to the needle insertion orientation. Bolus are highlighted in red post-injection and indicated by an arrow at later time points.

Representative ultrasound images of the injection site from each treatment group are presented in Figure 2. The images were similar regardless of the injection flow rate. Full imaging along the sagittal and transverse planes is presented in Figure S3. The test item was confirmed as being properly injected into the subcutaneous space, except for one depot of 0.2 mL injected at the fast flow rate, which was partially injected intramuscularly. These data were excluded from further analysis.

For each injection, the depot is predominantly fusiform and hypoechoic immediately after injection. From 24 h onward, an increase in echogenicity was observed, which is characteristic of ISFD phase inverting systems [30].

**Figure 3:**
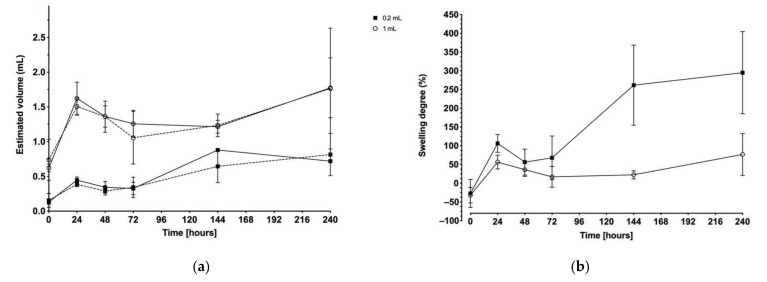
Variation of depot volume over the course of the study (10 days): (**a**) the calculated volumes from both injections are displayed at both injection sites: site 1 as full line; site 2 as dotted line; (**b**) the degree of depot swelling is presented for both volumes as the mean of the two injection sites.

Dimensions of the depot were measured from both sagittal and transverse depot images. The depot volumes at each injection site are presented in Figure 3a. With both 0.2 and 1 mL injection volumes, a sudden volume increase was observed at 24 h post-injection followed by a steady decrease down to the initial injected volume at 72 h post-injection. From this time point, the depots progressively expanded again. There was no significant difference between the measurements from the injection sites at two different injection flow rates (*p* = 0.9824, *n* = 73).

In Figure 3b, depots degree of swelling is presented as the mean of both injection sites (i.e., site 1 injected at 1 mL/min and site 2 injected at 10 mL/min). At 24 h post-injection, the mean swelling was +106% for a 0.2 mL test item injection and +57% for a 1 mL test item injection, representing an estimated volume of 0.4 and 1.6 mL, respectively. Maximum swelling was recorded at 240 h post-injection. For the 0.2 mL test item injection volume, a swelling ratio of +295% was reached corresponding to an approximate volume of 0.8 mL, an expansion of about four times the initial volume after 10 days. In parallel, the 1 mL test item injection volume swelled up to approximately 1.8 mL (+77% swelling degree), which is not significantly different from the 24 h data.

### 2.5. Histopathologic Evaluation

Injection sites were collected at either 72 h or 240 h post-injection to evaluate the short and longer-term evolution of a potential loco-regional reaction triggered by the injection procedure. The 72-h time point was appropriate to observe any acute inflammatory reaction related to the injection procedure, while the 240-h time point allowed to characterize the chronic inflammatory reaction produced by the depot.

**Figure 4:**
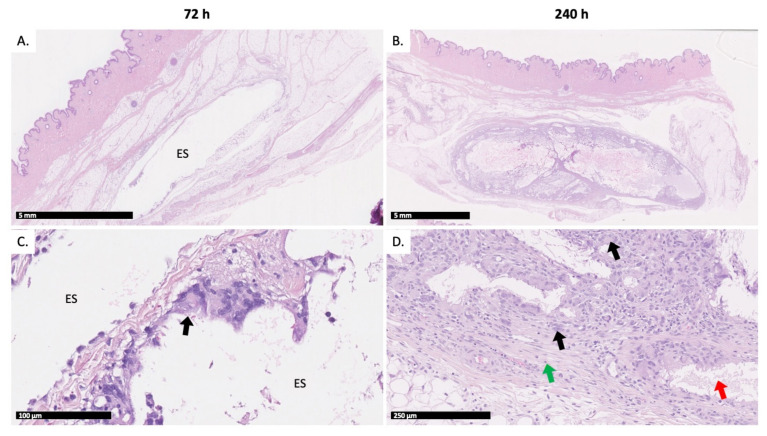
Representative H&E histopathology of injection sites: (**A**) test item injection site at 72 h and (**B**) test item injection site at 240 h. A focus on the fibrous capsule is presented (**C**) at 72 h, and (**D**) at 240 h. ES: poorly defined empty spaces in the subcutis. Green arrow: fibrous capsule. Black arrow: inflammatory cells, including multinucleated macrophages. Red arrow: empty spaces.

Animals 1 and 2 (Group 1) and 5 and 6 (Group 2) were sacrificed at 72 h post-injection. The remaining animals were sacrificed at 240 h. All histopathological findings were observed in the subcutis. Only one injection in animal 4 (0.2 mL test item treatment injected at 10 mL/min) was partially located in the abdominal wall. As the general shape of the depot was similar to other depots, it was not excluded from the microscopic evaluation. Representative histopathology slides are compiled in Figure 4.

At 72 h post-injection, a “pseudocyst” was observed in the SC space of seven of the eight investigated injection sites (Figure 4A) for both 0.2 and 1 mL injections. The pseudocyst or inflammatory cyst was a well-circumscribed cystic space with no epithelial lining marked out by inflammatory elements such as fibrosis or inflammatory cells. The observed empty spaces presumably originated from the test item dissolution during histology processing. At 72 h post-injection, pseudocysts were limited by a thin fibrous capsule admixed with few inflammatory cells, mainly macrophages, and the occasional presence of multinucleated macrophages (Figure 4C). In this connective tissue surrounding the cavities or the pseudocysts, various combinations of mixed inflammatory cell infiltrates, hemorrhage and/or necrosis were occasionally observed, mainly in animals that had received 1 mL test item injections. The severity of these changes was low (from minimal to mild). All changes at 72 h post-injection were observed independently of the injection flow rate and/or the injected volume.

At the 240-h time point, all animals had a well-defined subcutaneous encapsulated, often multilocular, nodule. This nodule was composed of empty spaces limited by fibrous cords combined with mixed inflammatory cells, among which numerous multinucleated giant macrophages were observed. Overall, these nodules are considered to be areas of granulomatous inflammation. In one instance (0.2 mL test item injected slowly), chronic inflammation was observed in the surrounding connective tissue. The severity of these changes was graded as moderate for most injection sites, and as marked on one occasion.

**Figure 5:**
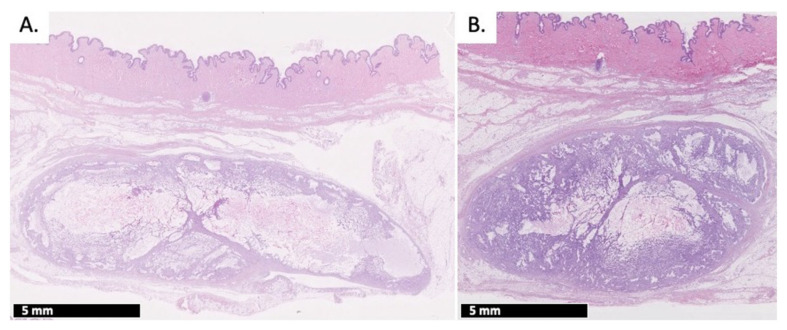
H&E histopathology of 1 mL test item depots at 240 h, administered at (**A**) slow and (**B**) fast injection flow rate.

Overall, reactions were similar in nature between animals treated with 0.2 mL and 1 mL of test item. Similarly, the rate of administration had no influence on the nature and severity of the loco-regional changes, as shown in Figure 5.

Full histopathology grading is available in Table S1.

## 3. Discussion

The ISFD BEPO^®^ technology is based on the combination of a drug substance with an injectable vehicle solution made of a diblock and a triblock PEG–PLA copolymer solubilized in an organic solvent. This long acting injectable technology forms a solid depot in situ allowing sustained delivery of an API over several days to several months [21]. The aim of the present study was to evaluate the influence of the injection flow rate and injection volume of a BEPO^®^ vehicle on the loco-regional skin tolerance.

Currently, little is known regarding the loco-regional tolerability of ISFD in the subcutaneous environment [5,12]. Due to the large variety of ISFD (i.e., different formation mechanism, applications, polymeric and solvent components, route of administration), it is difficult to generalize the available results. DMSO is an FDA-approved pharmaceutical excipient for parenteral administration, including the subcutaneous route. As for the copolymers, the tolerability of PEG–PLA copolymers has already been described in subcutaneous [31,32] and intravenous [33,34] administrations. However, data are lacking regarding their use in ISFD by phase inversion. Most published studies focus on PLGA-based ISFD [12,35,36,37,38]. Nonetheless, more than the components of the ISFD technology, the procedure to conduct a preclinical loco-regional toxicity study is not yet standardized. The few studies with PLGA ISFD using DMSO as solvent for subcutaneous administration have been conducted in a variety of species, going from monkey [39] to rat [40]. In our case, we favored the use of Göttingen minipig to assess subcutaneous reactions as this species has the closest resemblance to human subcutis [24,41]. Moreover, to the best of our knowledge, the administration procedure of ISFD has not yet been investigated. This lack of harmonized methodologies and historical data is showing the need for a tolerability study for a specific copolymer and solvent combination and its associated route and procedure of administration. In the present study, we decided to inject a highly viscous test item (>600 mPa.s) in the subcutaneous space of Göttingen minipigs using two different injection flow rates (1 and 10 mL/min) and volumes (0.2 mL and 1 mL), allowing to screen challenging parameters.

The endotoxin levels within the test item complied with the USP-calculated limit based on the highest injected volume (i.e., 1 mL). This result confirmed the low pyrogenic potential of the prepared product. In parallel, the in vitro characterization of the test item demonstrated that the highest targeted injection flow rate (10 mL/min) was consistent with an acceptable range of force required for manual injection in vivo (i.e., <20 N [29]) with the selected device (i.e., 1 mL Luer Lock Soft-Ject syringe equipped with 21G 5/8” needle). However, by reaching the limit of a comfortable manual injection, the probability of deviation from the targeted injection flow rate was increased. To comply with a 10 mL/min injection, the operator had to complete the administration in a short amount of time (i.e., within one second for a 0.2 mL injection and six seconds for a 1 mL injection) while being confronted with an important break-loose force. The observed deviation at 10 mL/h for the 0.2 mL injection was of −44%, with injection durations ranging from 1 to 3 s, while the one for the 1 mL injection was of −20%, with injections within 6 to 8 s. Therefore, the highest manual injection rate that a well-trained operator could achieve during this animal study was up to 8 mL/min instead of 10 mL/min.

From a macroscopic point of view, all injections were well tolerated regardless of the injection volume and flow rate. Indurations were noticed throughout the 10-day study. Because of the very thin skin and hypodermis at the plica inguinalis site, the presence of the depots was evident upon palpation, suggesting that the induration mainly resulted from the polymeric precipitate formed in the SC space. In addition, ultrasound images allowed the confirmation of the presence of a hyperechoic structure (i.e., the depot), with a volume evolving similarly to the induration recorded, supporting the observation that the local response was mainly linked to the presence of the polymeric depot.

Ultrasound imaging was performed and confirmed that the injections were properly delivered into the subcutaneous tissue. From a morphological point of view, the injected test item formed an oblate ellipsoid depot upon precipitation that stretched along the sagittal axis (i.e., a similar direction to needle insertion) and remained visible throughout the 10-day study. The presence of the depot at the last time point was expected because of the specific polymeric composition of the tested BEPO^®^ vehicle. These observations also confirm the relevance of the ellipsoid volume formula to determine the depot volume. With ultrasound imaging, the injected test item was hypoechoic immediately after injection and for the first 24 h, suggesting the presence of liquid within the depot (e.g., solvent, fluids from the tissues or mixture both). Overall, the depot morphology was similar with slow and fast injection flow rates. Qualitatively, the depot became more hyperechoic 48 h post-injection. According to the literature, this change in echogenicity reflects the copolymer precipitation during the depot formation [30,42,43].

Measurements of the depot dimensions along the sagittal and longitudinal plains were expected to be a direct measurement of the formed depot. Depot swelling was observed with both 0.2 and 1 mL injection volumes and peaked at 24 h and 240 h post-injection, regardless of the injection flow rate. Swelling is a common feature of ISFD, especially due to the water uptake upon solvent exchange [44,45,46]. In addition, BEPO^®^ depots are known to often exhibit a porous internal structure [21] characteristic of a rapid solvent exchange process. Consequently, they might be prone to fluid uptake due to the more or less interconnected network of pores. Moreover, the extent and kinetics of swelling are suspected to depend on the polymeric composition of the depot, as it has previously been highlighted in works on polyester polymers [42,46]. In consequence, the first volume increase (i.e., at 24 h) could be associated to the fast solvent/non-solvent phase exchange while the progressive expansion up to 240 h could be the result of the particular polymeric composition exploited in this study combined with a porous internal network characteristic of BEPO^®^-based products. Interestingly, the depots formed with lower injection volumes (i.e., 0.2 mL) tended to expand more than those with an injection volume of 1 mL, proportionally to the initial volume injected. At the later time point (i.e., 240 h post-injection), 1 mL depots swelled up to 1.8 times their initial volume. In contrast, 0.2 mL depots swelled more with a 4-fold increase in volume. A potential explanation for this volume-dependent swelling behavior could be that a maximum local expansion of the subcutaneous environment surrounding the depot was reached with the higher volume. This mechanical restriction would thus allow for a greater expansion of the smaller than the larger volume.

To complete this study, histopathological examination of the injection sites was performed at either 72 h or 240 h post-injection, to evaluate the short and longer-term evolution of a potential loco-regional reaction, while assessing the impact of the injection flow rate. Pseudocysts were observed at 72 h post-injection at both injection sites, whereas nodules were recorded at 240 h post-injection. In both morphological changes, empty spaces were observed: unique and large cavities in the case of pseudocysts, smaller and multilocular cavities in the case of nodules. These cavities most probably represent the location of the polymeric depots, which dissolved during tissue processing, hence their empty appearance. At early time points, only a few multinucleated macrophages were observed within the pseudocyst. Later, the cavity was gradually replaced by a mature granulomatous inflammation comprising fibrosis, inflammatory cells and many multinucleated giant macrophages. These pseudocysts and nodules represent a continuum characteristic of an expected foreign body reaction (FBR) [47,48]. FBR is an innate biological response taking place in two phases: (1) an acute phase for immediate injury response lasting from hours to days with blood and tissue fluid proteins accumulating around the foreign material and migration of neutrophils and (2) a chronic phase with macrophage proliferation for foreign material engulfment, creation of foreign body giant cells (FBGC), and proliferation of fibroblasts to form a fibrous capsule around the depot [14,49,50]. In this study, fibrosis was recorded from 72 h post-injection, which means that the FBR chronic phase response was already active. It can be suspected that the acute phase was initiated immediately after injection. It can also be supposed that the involved fluid accumulation in this phase accentuated the early swelling behavior observed at 24 h. At 240 h post-injection, the non-negligible presence of multinucleated giant macrophages, i.e., FBGC, confirms that macrophages have entered a “frustrated phagocytosis” phase, as they are unable to degrade the depot individually [47,48,51]. This prolonged FBR was expected given the particular polymeric composition of the test item. As a matter of fact, the vehicle was designed to represent a slow degradation rate (i.e., it had a high polymeric content and copolymers with a long hydrophobic chain length).

## 4. Materials and Methods

### 4.1. Materials

Diblock mPEG-PDLLA and triblock PDLLA-PEG-PDLLA copolymers were synthesized by CM Biomaterials (Tucker, GA, USA). USP grade DMSO (Procipient^®^) was purchased from Gaylord Chemical (Los Angeles, CA, USA). The diblock used in this study was composed of mPEG of 2 kDa and PDLLA of 9.8 kDa average molecular weight. The triblock used was composed of PEG of 1 kDa and PDLLA of 9.8 kDa. The in vivo study was conducted by CitoxLab Scantox A/S (Lille Skensved, Denmark) in compliance with the ARRIVE guidelines [52] and EU Directive 2010/63/EU for animal experiments.

### 4.2. Preparation of the Test Item

The BEPO^®^ vehicle was prepared by weighing the necessary amounts of TB and DB copolymers to reach a final concentration of 40% (weight/weight) in a glass vial, with a 1:1 TB:DB ratio. After addition of the proper amount of DMSO, the vial was sealed and left under agitation on a roller mixer at room temperature. Complete dissolution of the copolymers was assessed visually by obtaining a clear, translucent and viscous solution. The solution was then sterilized by filtration through a 0.2 µm PTFE filter, and immediately aliquoted into sealed clear sterile vials.

For the in vivo phase, one aliquot of each test item was sent to CitoxLab Scantox A/S at +2–8 °C. Another aliquot was kept at MedinCell labs (Jacou, France) for characterization.

### 4.3. Characterization of the Test Item

#### 4.3.1. Dynamic Viscosity Determination

Test item dynamic viscosity was determined using a Rheometer MCR301 (Anton Paar GmbH, Graz, Austria) associated with a Peltier temperature system (P-PTD 200, Anton Paar GmbH, Graz, Austria) connected to the Rheocompass v1.25.422 software (Anton Paar GmbH, Graz, Austria). The measuring system used was a Cone-Plate of 50 mm diameter and 1° angle (CP50-1, truncation: 104 µm). Approximately 700 µL of product was poured onto the Peltier plate before lowering the measuring system. Measurements were performed using a rotational method at +25 °C. The shear rate was controlled from 10 s^−1^ to 1000 s^−1^ in a logarithmic ramp, with 10 points/decade and 5 s per point.

#### 4.3.2. Assessment of Injectability

The injection forces required at the targeted flow rates of 1 and 10 mL/min were determined by using a Texturometer FTPlus friction tester (Ametek STC, Berwyn, PA, USA) piloted by the NEXYGENPlus 3.0 software (Ametek STC, Berwyn, PA, USA). Flow rates were set as follows: 10 mL/min was found to be the fastest achievable manual injection speed from tests performed by several technicians; 1 mL/min was considered the slowest acceptable flow rate for an injection duration compliant with the animal well-being and study procedure, taking into account the volumes to be administered. Tests were performed with the same syringe, needle type and brand as those used during the in vivo administration (i.e., 1 mL Luer Lock Soft-Ject syringe equipped with 21G 5/8” needle). An excess of test item was filled into the syringe before locking the needle. Priming was performed by adjusting the injection volume to 500 µL. A compression method was used at room temperature (+20 °C ± 5) with a preload stress of 0.2 N. Test speed was calculated according to the syringe tube length, to convert the targeted flow rate from mL/min to mm/min. The full force profile for the test item delivery at each injection flow rate was recorded using the pressure sensor of the Texturometer. The injection force was determined as the mean sustained force applied to the plunger to expel the test item from the device.

#### 4.3.3. Bacterial Endotoxins Test

Bacterial endotoxins were dosed in the pre-clinical batch to assess the pyrogenicity of the product. Quantification was performed by Nelson Labs (Heverlee, Belgium) by dissolution of 200 mg of product into 1 mL of Acetonitrile. The test article was assayed in a microtiter plate in duplicate, at a 100-fold dilution in Limulus amoebocyte lysate (LAL) reagent water. The microtiter plate was pre-incubated in a plate reader at 37 ± 1 °C for ≥ 10 min. After incubation, kinetic QCL-reagent (0.1 mL) was added to each well, and the absorbance at 405 nm was assessed and recorded every 150 s for a total of 40 data points, or until the concentration reached 0.2 absorbance units. The experimental value was determined using a standards curve that was prepared in parallel.

According to USP chapter <85> bacterial endotoxins test (BET) [53], endotoxin limit was calculated as

Endotoxin limit = K/M,(1)

with K being the threshold human pyrogenic dose per kg of body weight to be administered parenterally (=5 EU/kg) and M the maximum recommended bolus dose to be administered by kg of body weight. In this study, M equaled to 1 mL/10 kg (estimate 4–5 months old minipig body weight), leading to an endotoxin limit of 50 EU/mL.

### 4.4. In Vivo Study Design

Two volumes of the test item were administered to assess the influence of the injection volume on injection site tolerability. A volume of 0.2 mL was considered the lowest amount to be manually filled into a 1 mL syringe with sufficient precision. A volume of 1 mL was set as the highest injectable volume, using the same injection device.

#### 4.4.1. Injection Site Selection

**Figure 6:**
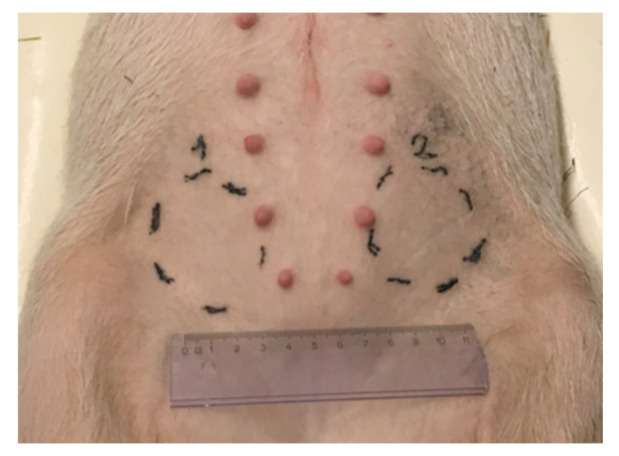
Marking of injection sites in the plica inguinalis of a minipig.

The study was performed in 8 female Göttingen SPF minipigs purchased from Ellegaard Göttingen Minipigs A/S (Dalmose, Denmark). The age of the animals ranged from 4 to 5 months old, with a body weight of ca. 10 kg at arrival. The plica inguinalis was selected as the subcutaneous injection site because of known similarities with human skin in terms of thickness and the lack of panniculus carnosus, resulting in similar mechanical constraints [23,54,55]. Injection sites were tattooed one week before dosing as displayed in Figure 6.

Animals were randomized in 2 groups of 4 animals:

- Group 1: 0.2 mL of test item (Animals #1 to #4);
- Group 2: 1 mL of test item (Animals #5 to #8).

To limit inter-animal variability, each animal received two injections of the same dose, at different flow rates:

- Site 1: target 1 mL/min injection flow rate;
- Site 2: target 10 mL/min injection flow rate.

The animals were sacrificed at either 72 h (first two animals from each group) or 240 h (last two animals from each group) post-injection, and injection sites were recovered for further analysis.

#### 4.4.2. Injection of the Test Item

The test item was supplied as ready-to-use. Samples were removed from the fridge and kept at room temperature the day before dosing to ensure that test item had completely thawed before injection (DMSO melting point = +18–19 °C). Animals were injected under general anesthesia with intramuscular ketamine (6.25 mg/kg of 50 mg/mL) and midazolam (1.25 mg/kg of 5 mg/mL) to maximize the consistency and reproducibility of injections, ketamine and midazolam being a common cocktail used as premedication of Göttingen minipigs. The test item was injected subcutaneously using a 1 mL Luer Lock Soft-Ject syringe (Henke Sass Wolf GmbH, Tuttlingen, Germany) equipped with a 21G 5/8” needle (BD Microlance, Becton Dickinson, Franklin Lakes, NJ, USA). The skin was pinched, and the full length of the needle was inserted with an angle between 45° and 90°. Injection duration was recorded using a timer to calculate the experimental injection flow rate at each injection site. After each injection, the needle was left under the skin for 5 s before removal to minimize leakage of the product. On the day of necropsy, the animals were examined externally and then anaesthetized by an intramuscular injection (about 0.3 mL/kg), with a mixture of Zoletil 50 Vet. (Virbac S.A., Carros, France), 20 mg xylazine/mL (6.25 mL), 100 mg ketamine/mL (1.25 mL) and 10 mg butorphanol/mL (2.5 mL). The animals were sacrificed by exsanguination and the injection sites explanted for histopathology analysis.

### 4.5. Assessment of Loco-Regional Skin Tolerance

Macroscopic observations were performed daily on unanesthetized animals laying on their backs, allowing a scoring of the injection sites. Loco-regional reactions, in particular hematoma, erythema, swelling such as oedema (soft swelling) or induration (hard swelling), scab, wound and scar, were recorded. Palpation was only allowed beyond 24 h post-injection to avoid interferences with the depot formation. On the day of dosing, the observations were recorded before and immediately after the injection.

### 4.6. Ultrasound Imaging

Ultrasound imaging was performed using a LOGIQ E9 (GE Healthcare, Chicago, IL, USA) with an ML6-15 transducer in B-mode at a frequency ranging from 13 to 15 MHz. Scanning was performed both pre- and post-injection, and then subsequently at 24 h, 48 h, 72 h, 144 h and 240 h post-injection. Non-sedated animals were scanned from 24 h post-injection until study termination. Imaging was performed along the sagittal and transverse planes to allow for measurements of the depot. Measurements were collected using Horos™ v3.3.6 software (Horos Project, New York, NY, USA). Depot volume was estimated using the ellipsoid volume formula:

V_depot_ = π/6 × length × width × height, (2)

Swelling degree (%) was also determined using the formula:

Swelling degree = (V_depot_ − V_injected_)/V_injected_ × 100%, (3)

Findings were interpreted by two independent experts in ultrasound imaging.

### 4.7. Histopathologic Evaluation

Injection sites (depots and surrounding tissues) were entirely explanted at either 72 h or 240 h post-injection and prepared for histological processing. Skin samples were fixed in 10% buffered formalin, embedded in paraffin and cut at a nominal thickness of approximately 5 μm, stained with Hematoxylin and Eosin (H&E) and then examined under a light microscope. Histological alterations were graded using a 5-level scale (minimal, mild, moderate, marked and severe).

### 4.8. Statistical Treatment

All data are reported as a mean with standard deviation. Statistical significance of the different data sets (*p* < ⍺ = 0.05) was also investigated. Unpaired t-test and one-way analysis of variance (one-way ANOVA) were used for mean data comparison of injectabilities and ultrasound measurements comparison, respectively. Statistical treatment was performed using XLSTAT software (Addinsoft, Bordeaux, France).

## 5. Conclusions

This in vivo study in Göttingen minipigs allowed the demonstration of the loco-regional safety of a test item based on ISFD BEPO^®^ technology when injected subcutaneously at different flow rates and volumes. Injection site tolerability of the depot was evaluated by macroscopic and histopathological examination of the injection sites. Ultrasound imaging was also performed to support macroscopic observations and allow longitudinal depot measurements. Together, these experiments showed an acceptable loco-regional response to the injected polymeric test item, designed to be an extreme case study (i.e., a vehicle of very high viscosity and slow degradation kinetics), following low as well as high injection flow rates. The formation of a well-circumscribed subcutaneous nodule was observed along with a typical FBR for all injections. The scoring of the injection site reactions was volume-dependent and no cases of treatment-emergent adverse events were reported, ruling out any matter of concern from a histopathological perspective. It can thus be considered that the ISFD PEG–PDLLA based-product is well tolerated when injected subcutaneously at volumes ranging from 0.2 mL to 1 mL and injection flow rates up to 8 mL/min.

Based on the loco-regional tolerance data noted in the Göttingen minipigs, a human-relevant animal model for subcutaneous administration, we consider the ISFD BEPO^®^ technology likely to be safe in human patients.

## Figures and Tables

**Table 1:** Test item characterization results.

H	Viscosity	Injection Force		Endotoxin Level
		1 mL/min Flow Rate	10 mL/min Flow Rate	
Test item	625 mPa.s (5) *	2.5 N (0.6) *	19.0 N (0.2) *	<2.339 EU/mL

* Standard deviation.

**Table 2:** Experimental injection flow rates. Deviations were calculated from the targeted injection flow rates (i.e., 1 mL/min or 10 mL/min).

	Target 1 mL/min		Target 10 mL/min	
	Flow Rate	Deviation	Flow Rate	Deviation
Group 1–0.2 mL	1.0 mL/min (0.0) *	−2%	5.6 mL/min (1.4) *	−44%
Group 2–1 mL	1.0 mL/min (0.0) *	−2%	8.0 mL/min (1.0) *	−20%

* Standard deviation.

## Data Availability

The data presented in this study are available on request from the corresponding author. The data are not publicly available due to commercial restrictions.

## Supplementary Materials

The following are available online at https://www.mdpi.com/article/10.3390/ijms22179250/s1.
