# Supplementary material for: Evaluation of Loco-Regional Skin Toxicity Induced by an In Situ Forming Depot after a Single Subcutaneous Injection at Different Volumes and Flow Rates in Göttingen Minipigs
Source: Int J Mol Sci. 2021 Aug 26;22(17):9250. doi: 10.3390/ijms22179250 (PMC8431084; doi:10.3390/ijms22179250)
Supplement: Supplementary file 1 [file ijms-22-09250-s001.zip › ijms-1330510-supplementary.pdf]

ELECTRONIC SUPPLEMENTARY INFORMATION

Evaluation of loco-regional skin toxicity induced by an *in situ* forming depot after a single subcutaneous injection at different volumes and flow rates in Göttingen minipigs

Charlotte Peloso[1], Anne-Pascale Trichet[1], Jacques Descotes[2], Joël Richard[1], Christophe Roberge[1] and Adolfo Lopez-Noriega[1]*

*Corresponding author: adolfo.lopeznoriega@medincell.com

[1]MedinCell, 3 rue des frères Lumière, 34830 Jacou, France

[2] ImmunoSafe Consultance, 120 Chemin des Bouleaux, 38480 Saint Jean d'Avelanne, France

Figure S 1: Viscosity behavior of the test item at batch characterization (n = 3).

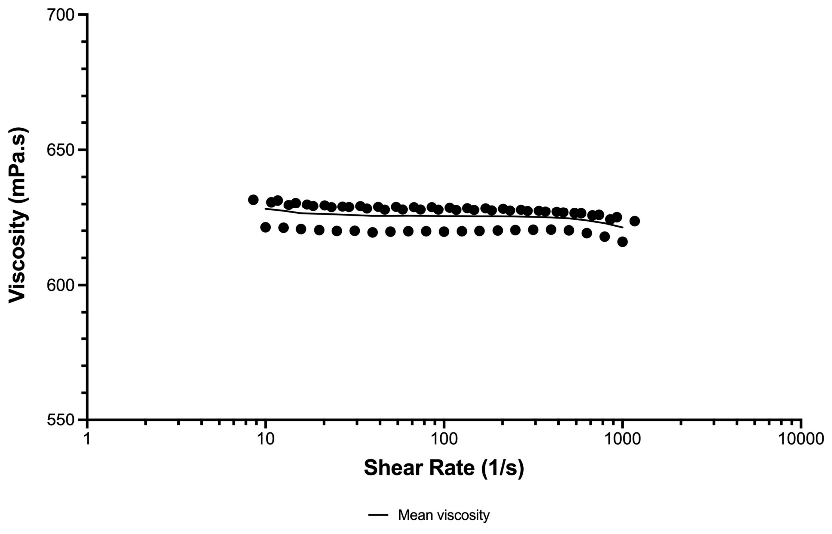

Figure S 2: Representative images of injection sites from the 0.2 mL treated group along the study

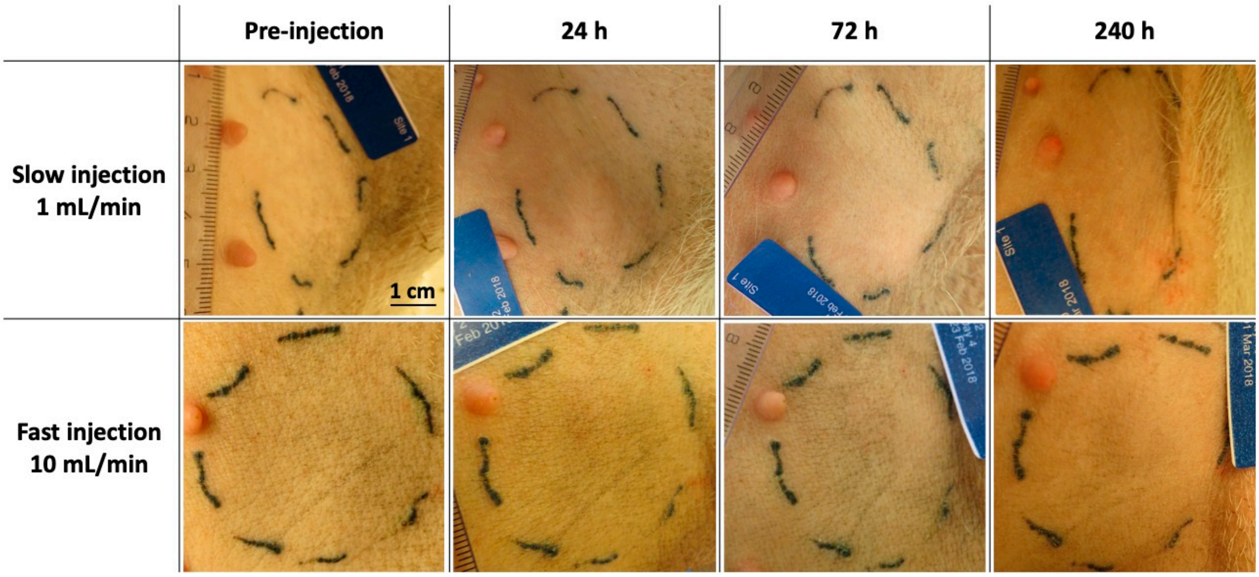

Figure S 3: Representative ultrasound images of A) 0.2 mL test item and B) 1 mL test item injection over the course of the study. Images were acquired along the transverse (1) sagittal (2) planes, at frequencies ranging from 13 to 15 MHz. Bolus (*i.e.* depot) are highlighted in red post-injection and indicated by an arrow at later time points.

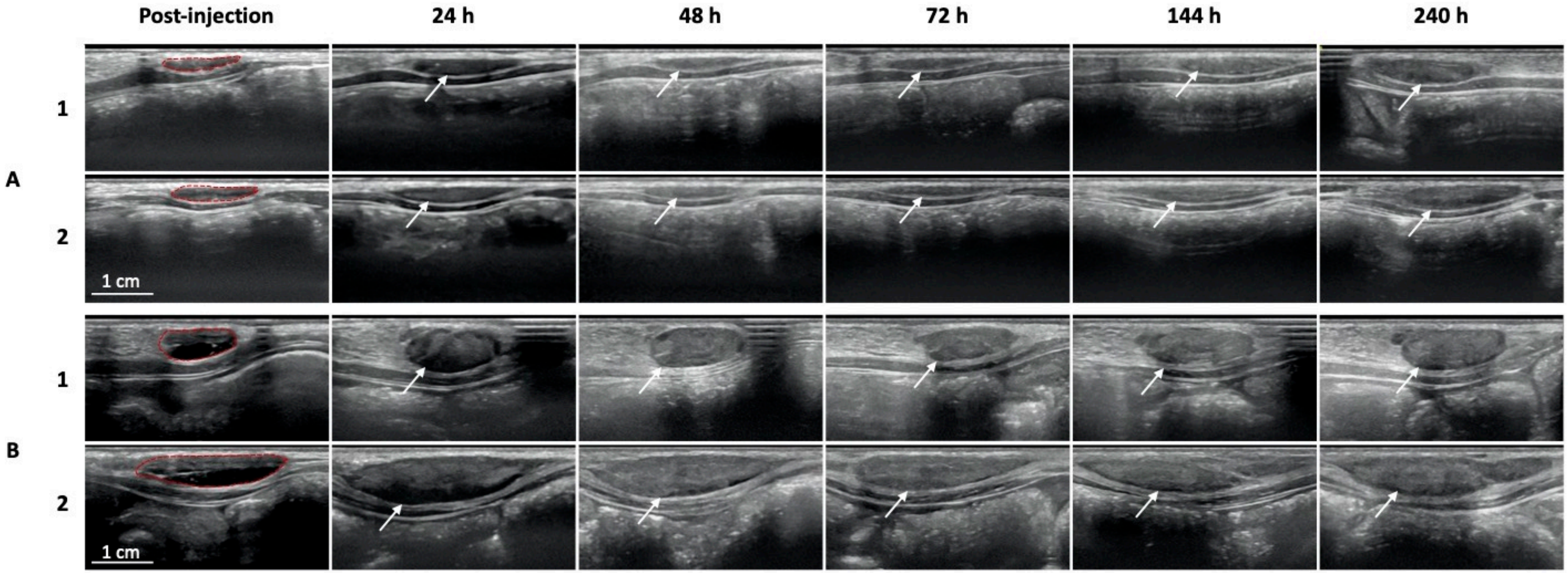

Table S 1: Histopathology grading of each injection sites involved in the study. F: focal; M: multifocal; D: diffuse; P: present. 1: minimal; 2: mild; 3: moderate; 4: marked.

Treatment	Necropsy time point	Animal #	Injection flow rate (mL/min)	Cavity	Pseudocyst	Nodule	Mixed inflammatory cell infiltration	FBGC	Hemorrhage	Necrosis	Chronic inflammation
Group 1 0.2 mL test item	72 h	1	1	P							
			10		P			1			
		2	1		P			1			
			10		P		2M	1			
	240 h	3	1			P		3			3D
			10			P		3			
		4	1			P		3			
			10			P		3			
Group 2 1 mL test item	72 h	5	1		P		1D		1M		
			10		P		2D				
		6	1		P		2D	1		1F	
			10		P		1D		1M		
	240 h	7	1			P		3			
			10			P		3			
		8	1			P		4			
			10			P		3			
